# When the body speaks of loss: psychosomatic dysregulation as a mediator between insecure attachment and prolonged grief symptoms in oncological and traumatic bereavement

**DOI:** 10.3389/fpsyg.2025.1708285

**Published:** 2025-11-13

**Authors:** Vittorio Lenzo, Sergio Triscari, Maria C. Quattropani, Valentina Bordino, Filomena Cibelli, Luigi Lombardo, Vincenzo Caretti, Adriano Schimmenti, Lucia Sideli

**Affiliations:** 1 Department of Educational Sciences, Section of Psychology, University of Catania, Catania, Italy; 2 U.O.S.D. Hospice San Cataldo, Azienda Sanitaria Provinciale Caltanissetta, Caltanissetta, Italy; 3 Centro di Cure Palliative, Hospice, Korian-Italian Hospital Group, Rome, Italy; 4 Centro di Cure Palliative, Hospice Fondazione Sanità e Ricerca, Rome, Italy; 5 Department of Human Science, LUMSA University, Rome, Italy; 6 Department of Human and Social Sciences, UKE—Kore University of Enna, Enna, Italy

**Keywords:** prolonged grief disorder, attachment, psychosomatic dysregulation, cancer, mediation analysis, oncology, trauma

## Abstract

**Background:**

This study investigated the role of psychosomatic dysregulation as a mediating factor in the association between attachment insecurity and prolonged grief symptoms.

**Methods:**

A cross-sectional design was used to survey 227 adults who had lost a loved one due to oncological (*n* = 138) or traumatic causes (*n* = 89) (74.0% female; age = 48.11 ± 11.53 years). The two groups did not differ significantly in terms of age, gender, marital status, or living arrangement. Participants completed the relationship questionnaire, the psychosomatic dysregulation inventory, and the traumatic grief inventory SR +.

**Results:**

The severity of prolonged grief symptoms did not significantly differ between groups, although participants in the oncological loss group reported slightly higher symptom severity compared to the traumatic loss group. The prevalence of probable PGD was similar between groups, with 13.8% of the oncological group and 10.1% of the traumatic group meeting diagnostic criteria. Results of mediation analysis showed that psychosomatic dysregulation partially mediated the relationship between attachment anxiety and prolonged grief symptoms, whereas no significant mediation was observed for attachment avoidance.

**Discussion:**

These findings point out the importance of addressing psychosomatic processes in individuals with high attachment anxiety, in line with the hyperactivation model of the anxious attachment system, to better understand and support their grief responses.

## 1 Introduction

### 1.1 Definition and prevalence of prolonged grief disorder

Prolonged grief disorder (PGD) is a pathological response to the loss of a loved one that was recently included in major diagnostic systems, such as the International Classification of Diseases (ICD-11) (World Health Organization, 2018) and the Diagnostic and Statistical Manual of Mental Disorders, Fifth Edition, Text Revision (DSM-5-TR) (American Psychiatric Association, 2022). According to these classifications, PGD is characterized by chronic (at least 6 months for ICD-11, at least 12 months for DSM-5 TR) and severe symptoms of separation distress (e.g., intense longing or/and persistent preoccupation for the deceased) along with cognitive, affective and behavioral symptoms (e.g., difficulty to accept the loss, emotional numbness, difficulty in engaging in social and leisure activities). These reactions exceed social and cultural norms for the patient’s context and cause significant disability and a need for clinical attention (World Health Organization, 2018; American Psychiatric Association, 2022).

Recent systematic reviews (Lundorff et al., 2017; Yuan et al., 2024) and empirical studies indicated a prevalence of PGD in bereaved adults of the general population ranging from 3 to 10% (Rosner et al., 2021; Shevlin et al., 2023; Treml et al., 2022), while the prevalence of any PGD symptoms (PGDS) is about threefold (Yuan et al., 2024). In Italy, characterized by a paucity of research on pathological grief, a recent study reported a prevalence of probable PGD of 7.7% among individuals who lost a loved one at least 12 months beforehand (Musetti et al., 2025). Certain populations seem to be at heightened risk for PGD (Rosner et al., 2021). Family caregivers of individuals with chronic and life-threatening illnesses, including neurological (Aoun et al., 2020; Crawley et al., 2023; Leonardi et al., 2012) and oncological diseases (Coelho et al., 2022; Sardella et al., 2023; Zordan et al., 2019), showed higher levels of PGD, respectively 20% (Schulz et al., 2006) and 14.2% (Kustanti et al., 2022). Similarly, individuals bereaved by sudden or violent losses, such as suicide, homicide, or accidents, tend to experience elevated rates of PGD, with around 49% affected (Djelantik et al., 2020). The difference in PGD rates between traumatic deaths and chronic illnesses has traditionally been explained in terms of anticipatory grief, the notion that in illness pathways, the possibility of “anticipating” the loss, in the case of chronic illness, may mitigate the intensity of bereavement, consequently reducing the psychopathological risk (Rogalla, 2020). However, as highlighted in the literature, the role of anticipatory grief is far from clear. Some studies have shown that, contrary to early hypotheses, high levels of pre-loss grief combined with poor preparation for bereavement are associated with worse, rather than better, post-loss outcomes (Nielsen et al., 2016).

Unfortunately, only a few studies have directly compared PGD or PGDS in individuals bereaved by chronic illness with those who lost a loved one to unnatural or traumatic causes (Cleiren et al., 1994; Miyabayashi and Yasuda, 2007). Moreover, no study has specifically focused on bereaved caregivers of cancer patients compared with individuals experiencing traumatic bereavement.

### 1.2 The contributory role of attachment insecurity and psychosomatic dysregulation

Attachment theory offers a meaningful framework for understanding individual differences in grief responses. Bowlby conceptualized attachment as an innate motivational system that drives humans to seek and maintain emotional closeness with caregivers and, later in adulthood, with others significant in their lives (Bowlby, 1969, 1988). The death of a close person can profoundly disrupt this system, compromising the mourner’s sense of safety and emotional regulation (Bowlby, 1980). Empirical research has highlighted two dimensions of attachment insecurity– attachment anxiety and avoidance—that originate in childhood and affect emotional responses and beliefs about the self and others throughout life (Ainsworth et al., 1978; Brennan et al., 1998; Hazan and Shaver, 1987). Individuals with attachment anxiety tend to frequently experience concerns that attachment figures may not be available in times of need. This dimension is often characterized by a negative self-image and heightened emotional sensitivity, leading to excessive dependence on others and difficulties in regulating emotions and bodily states (Mikulincer et al., 2003; Mikulincer and Shaver, 2019, 2022; Schimmenti and Caretti, 2018). Furthermore, attachment anxiety is associated with a higher risk of PGDS (Lai et al., 2015; Majd et al., 2024; Xu et al., 2015)^.^ By contrast, individuals with attachment avoidance tend to maintain emotional distance from others, inhibit their needs for care and feelings of vulnerability. They often express mistrust toward others’ intentions and tend to rely on self-reliant coping (Brennan et al., 1998). Although this strategy may offer short-term protection from emotional pain, it has been associated with physiological dysregulation and reduced engagement in adaptive coping mechanisms, potentially contributing to long-term psychopathological risk (Mikulincer and Shaver, 2019). However, meta-analytic evidence has not supported a direct association between attachment avoidance and PGDS (Buur et al., 2024). In this vein, some studies have proposed more complex explanatory models, suggesting that the effect of attachment avoidance on PGDS might emerge indirectly. For instance, a recent study found that attachment avoidance moderated the relationship between social support and PGDS, reducing the protective effect of social support (Lenzo et al., 2022).

In this framework, psychosomatic dysregulation may represent a key mechanism through which attachment insecurity may increase the risk for PGDS. The notion of psychosomatic dysregulation refers to a condition where difficulties in managing emotions and psychological states are expressed as physical symptoms or diseases that lack an objective medical cause (Schimmenti, 2017). Psychosomatic dysregulation denotes a disruption in the integration of bodily and affective processes, rooted in altered interoceptive and autonomic functioning, where emotional meaning fails to emerge from physiological states, leading to somatic expression rather than symbolic representation (Schimmenti, 2017). This condition is different from that of emotional dysregulation, which refers to the reduced ability to modulate, integrate, and express affective states within conscious experience and the social context and derives mainly from disorders in psychological regulatory networks. In brief, emotion dysregulation concerns the difficulty in managing emotions within awareness and relationships, whereas psychosomatic dysregulation concerns the failure to translate bodily states into emotional awareness, resulting in the body becoming the main stage of emotional communication (Schimmenti, 2017). Psychosomatic dysregulation is a cross-cutting mechanism that can occur in various clinical conditions, including apparently unrelated conditions like pediatric vasovagal syncope, where disturbances in autonomic and emotional regulation lead to syncopal episodes (Caretti et al., 2025). To stay on the topic of PGD, repeated experiences of insecure care can compromise the coordination between physiological activation and attribution of affective meaning (Maunder and Hunter, 2001). Over time, this can consolidate into a persistent tendency to communicate emotional needs somatically (Maunder and Hunter, 2001). Such a mode of operation, characterized by poor integration between systems, can hinder symbolic loss processing and impair adaptive pain regulation mechanisms, thus increasing vulnerability to PGD. A recent systematic review pointed out that PGDS are associated with somatic symptoms such as hypertension, insomnia, and other health concerns (Cunningham et al., 2025). Among bereaved family caregivers, sleep disturbances and impaired physical functioning reflecting wider difficulties in emotion regulation are common and persist long after the loss (Miller et al., 2020; Pohlkamp et al., 2019). Moreover, previous research consistently found that difficulties in emotion regulation and impaired reflective functioning (i.e., difficulties in understanding own and others’ mental states) were related to more severe PGDS (Giunta et al., 2024; Boelen et al., 2011; Lenferink et al., 2018; Milman et al., 2019). Despite these promising findings, research on the mediator role of psychosomatic dysregulation in the bereaved—particularly in those who lost a loved one to cancer or a traumatic accident—is still lacking. One study found that difficulties in recognizing emotions (i.e., alexithymia) were associated with PGDS (Lai et al., 2014). However, it remains unclear whether and how attachment insecurity may affect PGDS through psychosomatic dysregulation, a potential pathway that has received limited empirical attention to date.

**Figure 1:**
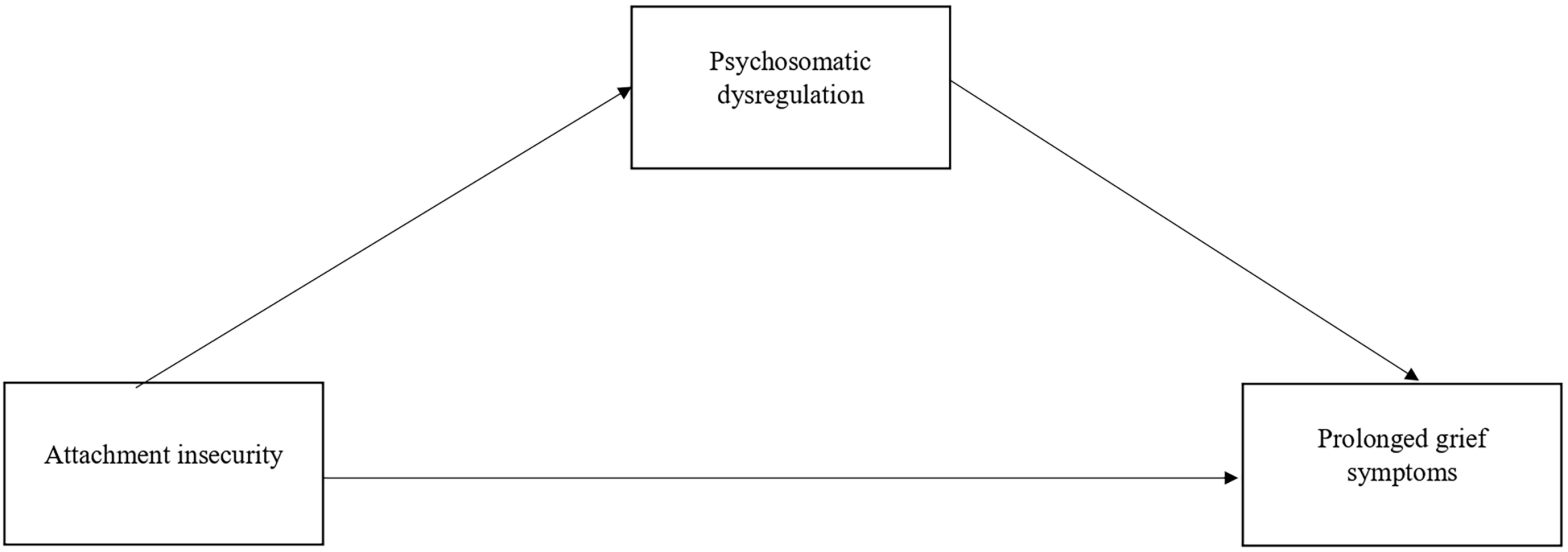
Model depicting the potential mediating role of psychosomatic dysregulation in the relationships between insecure attachment insecurity and the severity of prolonged grief symptoms.

Based on these premises, this study aimed to achieve two main objectives. First, we sought to compare the severity of PGDS and the prevalence of probable PGD between individuals who experienced bereavement due to oncological loss versus those who faced traumatic loss. We hypothesized that differences between these two groups would be smaller than traditionally assumed. Second, we aimed to explore the possible mediating role of psychosomatic dysregulation in the relationship between attachment styles and depressive symptoms. We hypothesized that psychosomatic dysregulation would mediate the relationship between insecure attachment styles and PGDS. The hypothesized model is illustrated in Figure 1.

## 2 Methods

### 2.1 Participants and procedures

The present study was part of a broader research project aiming at the Italian validation of the Traumatic Grief Inventory Self-Report Version Plus (TGI-SR+) (Lenferink et al., 2022). A cross-sectional observational design was adopted, comparing individuals bereaved by oncological or traumatic loss on psychological outcomes related to prolonged grief. Data was collected between July 2023 and August 2024. Participants in the oncological loss group were recruited through direct contact with coordinators of three Local Health Units of the Italian National Health Service (INHS) in Central and Southern Italy. These coordinators subsequently invited family caregivers of deceased end-of-life cancer patients to participate in the study. Inclusion criteria were age between 18 and 65 years and self-reported adequate fluency in the Italian language. Exclusion criteria included experiencing a loss within the past year and having a preexisting or current diagnosis of a severe mental disorder (e.g., bipolar disorder, psychosis) or dementia. Among the caregivers contacted, 156 agreed to participate on a voluntary basis. Two participants were excluded due to a preexisting or current severe mental disorder, while a further 16 participants were excluded because they were older than 65 years, resulting in a final sample of 138 participants. The comparison group (*n* = 89) was drawn from the sample involved in the Italian validation of the TGI-SR+, including individuals who had experienced a traumatic loss (e.g., accident, natural disaster, heart attack, suicide), and matching participants based on age and gender. Participants in this group were selected to match the oncological loss group in terms of age and gender. The study was conducted in accordance with the 1964 Helsinki Declaration and its subsequent amendments. Approval was obtained from the Ethics Review Board of Psychology Research of the University of Catania (Prot. n° Ierb-Edunict-2023.01.16/5). All participants provided informed consent prior to participation, and their privacy was protected in accordance with the European Union General Data Protection Regulation (EU GDPR 2016/679).

### 2.2 Measures

Demographic information comprised age, gender, educational background, and occupational status. Bereavement-related information included time since the loss, kinship relationship to the deceased, and cause of death. The following instruments were administered:

The Traumatic Grief Inventory Self-Report Version Plus (TGI-SR+) (Lenferink et al., 2022). The TGI-SR + is a self-report instrument to assess PGD according to DSM-5-TR criteria (American Psychiatric Association, 2022). It consists of 22 items rated on a 5-point Likert scale ranging from “Never” (World Health Organization, 2018) to “Always” (Rosner et al., 2021). Higher scores indicate a greater severity of PGDS, with each item reflecting a symptom experienced by the individual during the last year. To meet the DSM-5-TR criteria for PGD (American Psychiatric Association, 2022), participants should rate as “often” or “always” at least one out of two items assessing Criterion B (separation distress; items 1 and 3); at least three out of eight items assessing Criterion C (cognitive, emotional, and behavioral symptoms; items 6, 9, 10, 11, 18, 19, 21, and one between items 2 and 8); and the single item assessing Criterion D (functional impairment; item 13). The original version (Lenferink et al., 2022) demonstrated good internal consistency, with McDonald’s omega values of 0.92 for DSM-5-TR PGDS in both bereaved community samples and individuals who lost loved ones in traffic accidents. For probable DSM-5-TR PGD belonging, Lenferink and colleagues (Lenferink et al., 2022) identified the optimal cut-off score as ≥71 of the total score of the TGI-SR+. In the current study, the Italian version of the TGI-SR + (Lenzo et al., 2025; Sideli et al., 2022) showed excellent internal consistency with a McDonald’s omega value of 0.95 for both samples.

The relationship questionnaire (RQ) (Bartholomew and Horowitz, 1991). The RQ is a self-report instrument to assess four prototypical attachment styles, based on the positive or negative representations of self or others. Participants rate their agreement with four first-person statements, each corresponding to a specific attachment style, using a 7-point Likert scale ranging from 1 (“strongly disagree”) to 7 (“strongly agree”). The four prototypical attachment styles include: (a) secure attachment (RQ Secure), reflecting a positive view of both self and others; (b) dismissing attachment (RQ Dismissing), characterized by a positive view of self and a negative view of others; (c) preoccupied attachment (RQ Preoccupied), marked by a negative view of self and a positive view of others; and (d) fearful attachment (RQ Fearful), involving negative view of both self and others. In the present study, we employed the Italian version of the RQ, which has shown adequate psychometric properties (Carli, 1995). Following previous research (Brennan et al., 1998), two composite measures of attachment insecurity were calculated: attachment avoidance [(dismissing + fearful)—(secure + preoccupied)] and attachment anxiety [(preoccupied + fearful)—(secure + dismissing)].

The psychosomatic dysregulation inventory (PDI) (Caretti et al., 2019). The PDI consists of 101 items that assess various somatic symptoms using a 4-point Likert scale from 0 (“Never”) to 3 (“Very often or always”). Its development was informed by integrative perspectives combining neurobiological models of attachment with theories of psychosomatic functioning, drawing on work by Damasio (1996), Panksepp (1998), and Porges (2001). For this study, we used the short version of the PDI, which includes 20 items aimed at identifying risk for psychosomatic dysregulation. Specifically, this short version focuses on bodily symptoms and altered interoceptive experiences that indicate impairments in emotion recognition and regulation. This short form has demonstrated excellent internal consistency and good convergent and predictive validity in both clinical and nonclinical samples (Schimmenti, 2017). In the current study, the PDI showed excellent internal consistency with Cronbach’s alpha values of 0.90 for the oncological group and 0.93 for the traumatic group, respectively.

### 2.3 Statistical analysis

Statistical analyses were conducted using IBM SPSS Statistics version 29 and PROCESS macro for SPSS version 4.3 (Hayes, 2022). Descriptive statistics were computed for sociodemographic and bereavement-related variables. Group comparisons between participants who experienced an oncological loss and those who experienced a traumatic loss were conducted using independent samples *t*-tests for continuous variables and chi-square tests or Fisher’s exact tests for categorical variables. Two separate mediation analyses were conducted to examine the mediating role of psychosomatic dysregulation in the relationship between attachment insecurity dimensions (i.e., attachment anxiety and attachment avoidance) and the severity of PGDS. In each model, attachment anxiety and attachment avoidance were the independent variables, psychosomatic dysregulation was the mediator, and the severity of PGDS was the outcome variable. Type of loss (oncological vs. traumatic) and time since loss were included as covariates. The significance of indirect effects was evaluated using the bootstrapping method with 5,000 resamples and bias-corrected accelerated 95% confidence intervals (CIs) (Preacher and Hayes, 2004). Unstandardized regression coefficients (*B*), standardized coefficients (*β*), and standard errors (SE) were reported for all paths. An indirect effect was considered significant when the 95% confidence interval did not include zero. All statistical tests were two-tailed, and significance was set at *p* < 0.05.

## 3 Results

### 3.1 Characteristics of the participants

Table 1 shows the demographic and loss-related characteristics of the sample by oncological [*n* = 138] and traumatic group [*n* = 89]. The two groups did not significantly differ neither in age oncological loss group, 47.64 (*SD* = 12.01) vs. traumatic loss group, [48.94 years (*SD* = 10.89), *t* (225) = 0.44, *p* = 0.66], nor in gender distribution [74.6% female (*n* = 103) vs. 73.0% (*n* = 65), *χ*^2^(1) = 0.07, *p* = 0.79]. Moreover, no significant differences were found between groups regarding marital status, with the most frequent category being “married/cohabitant,” representing 44.2% [*n* = 61] of the oncological loss group and 53.9% [*n* = 48] of the traumatic loss group [*χ*^2^(2) = 3.39, *p* = 0.18]. No significant difference was also found in living arrangement, with most participants in both groups living with others [oncological loss group, 75.4% (*n* = 104) vs. traumatic loss group, 79.8% (*n* = 71), *χ*^2^(1) = 0.60, *p* = 0.44]. However, a significant difference was found between groups in terms of educational level, with participants in the traumatic loss group were more likely to have a graduate or post-graduate degree [64% (*n* = 57) vs. 45.7% (*n* = 63), *χ*^2^(1) = 7.35, *p* = 0.007]. Regarding loss-related characteristics, participants in the oncological loss group reported a significantly more recent loss compared to those in the traumatic loss group [*M* = 3.50 years, *SD* = 3.47 vs. *M* = 13.11 years, *SD* = 10.97, *t* (224) = −9.56, *p* < 0.001]. Furthermore, in the oncological loss group, most of the participants were sons or daughters of the deceased [66.7%, *n* = 92], whereas in the traumatic loss group, the distribution was more heterogeneous, with 44.9% [*n* = 40] losing an “Other” significant other (i.e., grandfather, cousin, mother-in-law) and 43.8% [*n* = 39] as sons or daughters.

**Table 1:** Demographic and loss-related characteristics of the sample by group.

Characteristics	Oncological loss (*n* = 138)	Traumatic loss (*n* = 89)	Statistic	*p*
Age, *M* (*SD*)	47.64 (12.01)	48.94 (10.89)	*t* (225) = 0.44	0.66
		73.0% (*n* = 65)		
Gender, % (*n*) female	74.6% (*n* = 103)		*χ*^2^(1) = 0.07	0.79
Education, % (*n*)			*χ*^2^(2) = 7.35	0.007
Middle or high school diploma	54.3% (*n* = 75)	36.0% (*n* = 32)		
Graduate or post-graduate	45.7% (*n* = 63)	64.0% (*n* = 57)		
Marital status, % (*n*)			*χ*^2^(2) = 3.39	0.18
Single	30.4% (*n* = 42)	30.3% (*n* = 27)		
Married/Cohabitant	44.2% (*n* = 61)	53.9% (*n* = 48)		
Separated/divorced/widowed	25.4% (*n* = 35)	15.7% (*n* = 14)		
Living arrangement, % (*n*)			*χ*^2^(1) = 0.60	0.44
Lives alone	24.6% (*n* = 34)	20.2% (*n* = 18)		
Lives with others	75.4% (*n* = 104)	79.8% (*n* = 71)		
Time since the loss, *M* (*SD*)	3.50 (3.47)	13.11 (10.97)	*t* (224) = −9.56	<0.001
Kinship to deceased, % (*n*)			FFH = 38.96	<0.001
Son/daughter	66.7% (*n* = 92)	43.8% (*n* = 39)		
Sibling	6.5% (*n* = 9)	6.7% (*n* = 6)		
Spouse	16.7% (*n* = 23)	4.5% (*n* = 4)		
Other	10.1% (*n* = 14)	44.9% (*n* = 40)		

*M*, mean; *SD*, standard deviation. Chi-square, *t*-test and Fisher–Freeman–Halton exact test statistics refer to between-group comparisons. There is one missing data for time since the loss in the oncological group.

### 3.2 Severity of PGDS and prevalence of probable diagnosis

The mean TGI-SR + total score did not significantly differ between the oncological and the traumatic group [55.13 (*SD* = 21.58) in vs. 49.87 (*SD* = 18.82), *t* (225) = 1.885, *p* = 0.061]. A probable diagnosis of PGD, defined in accordance with DSM-5-TR criteria operationalized through the TGI-SR + algorithm, was identified in 13.8% of the caregivers [*n* = 19] and in 10.1% of the individuals in the traumatic bereavement group [*n* = 9]. The difference in prevalence between the two groups was not statistically significant [*χ*^2^(1) = 0.669, *p* = 0.414], indicating a comparable proportion of individuals meeting criteria for probable PGD across the two samples.

### 3.3 Mediation analyses

**Figure 2:**
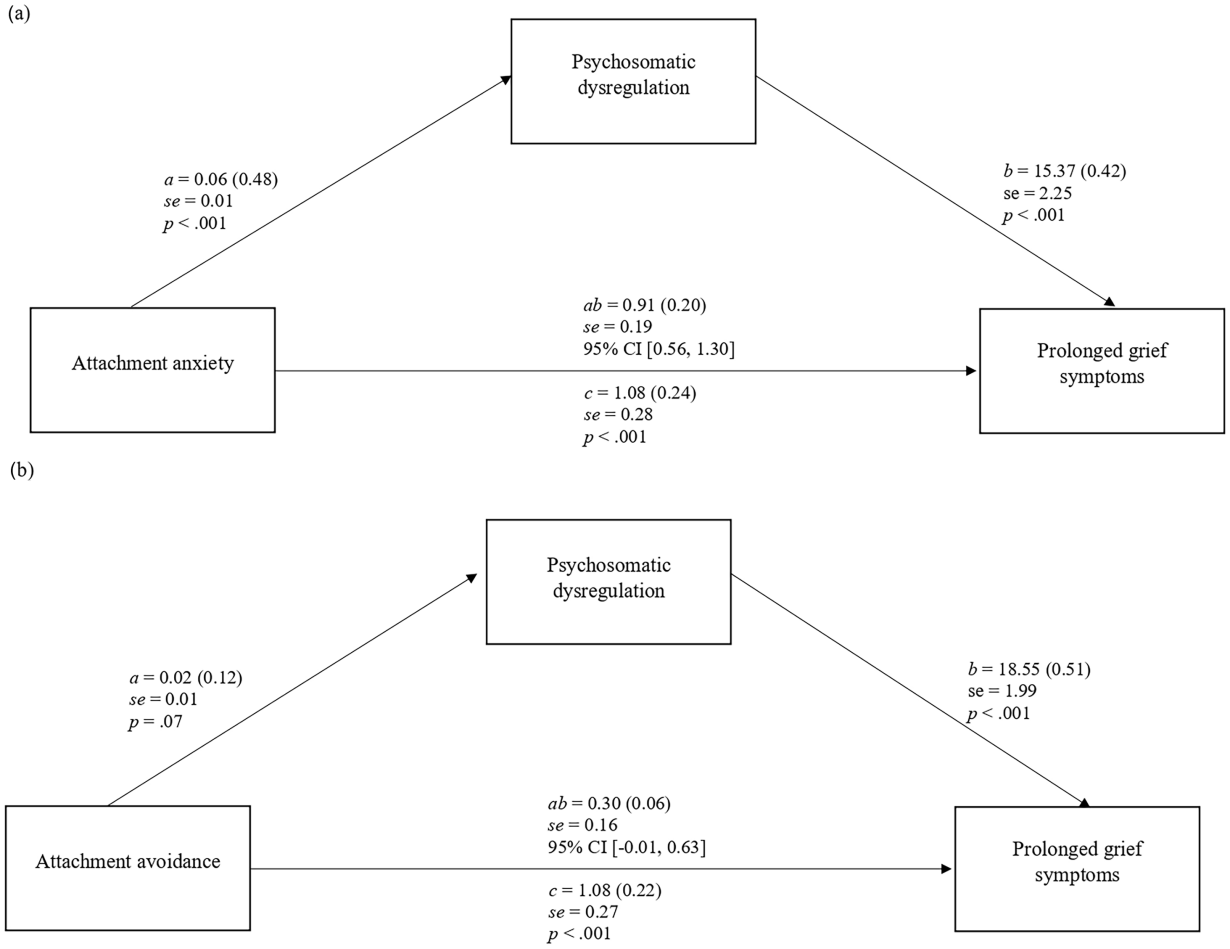
**(a,b)** Simple mediation model of attachment orientation on severity of prolonged grief symptoms through psychosomatic dysregulation. Values are unstandardized *B* coefficients (standardized coefficients are in parentheses). *SE*, standard error of *B. a*, effect of X on M; *b*, effect of M on Y; *c*, direct effect of X on Y; *ab*, indirect effect.

Figures 2a,b present the results of the mediation analyses. As shown in Figure 2a, attachment anxiety had a significant direct effect on PGDS [*b* = 1.08, *SE* = 0.28, *p* < 0.001, 95% CI (0.53, 1.63)], and psychosomatic dysregulation significantly mediated this relationship [indirect effect = 0.91, BootSE = 0.19, 95% CI (0.56, 1.30)]. Specifically, higher attachment anxiety was associated with greater psychosomatic dysregulation [*b* = 0.06, *SE* = 0.01, *p* < 0.001, 95% CI (0.04, 0.07)], which in turn predicted more severe PGDS [*b* = 15.37, *SE* = 2.25, *p* < 0.001, 95% CI (10.93, 19.80)]. The model reached a *R*^2^ of 0.35.

Regarding Figure 2b, attachment avoidance showed a significant direct effect on PGDS [*b* = 1.08, *SE* = 0.27, *p* < 0.001, 95% CI (0.54, 1.61)]. Psychosomatic dysregulation did not significantly mediate the relationship between attachment avoidance and prolonged grief [indirect effect = 0.30, BootSE = 0.16, 95% CI (−0.01, 0.63)]. Attachment avoidance was marginally associated with psychosomatic dysregulation [*b* = 0.02, *SE* = 0.01, *p* = 0.07, 95% CI (0.00, 0.03)]. The model achieved a *R*^2^ of 0.35. Type of loss (oncological vs. traumatic) and time since loss were included as covariates but did not show consistent significant effects in both models.

## 4 Discussion

To the best of our knowledge, this study is the first to compare PGDS in individuals who have lost a loved one due to an oncological condition with those who have experienced a loss from an immediate unnatural cause, such as suicide, heart attack, natural disaster, or accident.

According to our first hypothesis, the oncological loss group and the traumatic loss group did not show significant differences in the likelihood of receiving a diagnosis of PGD. Our findings suggest that the type of loss, whether anticipated through a long period, such as cancer, or sudden and unexpectedness, such as traumatic, may not entirely determine the likelihood of a diagnosis of PGD. Put another way, both types of bereavement may lead to persistent and maladaptive grief reactions. This finding is coherent with previous studies highlighting that cancer-related losses are associated with high levels of PGDS (Coelho et al., 2022; Sardella et al., 2023; Zordan et al., 2019). It is also consistent with recent findings on anticipatory grief, which highlight how prolonged caregiving under the burden of a loved one’s progressive decline can lead to maladaptive pain reactions following a loss (Nielsen et al., 2016). Together with the unexpectedness of traumatic grief, it should also be considered the prolonged stress related to a chronic disease among people who have lost their loved ones.

It is worth noting that those bereaved due to cancer in our study experienced a more recent loss compared to those bereaved due to a traumatic loss. Although some studies have found that a shorter time since loss is associated with higher symptom severity (Schwartz et al., 2018), others have found no significant relationship (Boelen and Lenferink, 2022). Our results contribute to this debate on the role of time during grief, suggesting that while it may shape symptom intensity to some extent, psychological mechanisms underlying grief may play a relevant role in determining who is at higher risk of PGD. It is therefore plausible that time since loss interacts with other individual and contextual variables, shaping the manifestation and persistence of grief symptoms rather than determining them in isolation. In this vein, the second aim was to investigate the mediating role of psychosomatic dysregulation in the relationship between insecure attachment orientations and the severity of PGDS. It is not surprising that individuals with high attachment anxiety tend to experience more severe grief reactions, as demonstrated by previous research (Lai et al., 2015; Xu et al., 2015; Majd et al., 2024). Indeed, attachment anxiety is characterized by hyperactivation strategies, including heightened vigilance to threats, distress when separated from significant others, and difficulties in regulating negative emotions (Mikulincer and Shaver, 2019, 2022). Our findings revealed that psychosomatic dysregulation had a mediating role in the path originating from attachment anxiety to PGDS. It is reasonable to hypothesize that it represents a key pathway through which attachment anxiety may lead to prolonged grief reactions. Difficulties in modulating emotions and bodily states may heighten somatic arousal, worsen sleep quality, and increase health complaints, which in turn reinforce distress associated with the loss, fostering a vicious cycle. These findings seem to be consistent with evidence that bereavement is often accompanied by bodily and somatic symptoms, and that difficulties in emotion regulation are associated with pathological grief severity. A different pattern emerged for attachment avoidance that requires further research. Our findings revealed a direct effect on PGDS, despite psychosomatic dysregulation did not mediate this association. Individuals with attachment avoidance tend to suppress attachment needs, maintain emotional distance, and rely on self-reliant coping strategies (Brennan et al., 1998). Psychosomatic dysregulation, indeed, involves difficulties perceiving internal states and sharing them in social contexts, including those related to loved ones. In the context of grief, these bereaved tend to deactivate their attachment system, thus often displaying a lack of awareness or minimization of their internal states in the relationship. However, this insecure attachment orientation does not protect them from prolonged PGDS, but it may outline other pathways to the disorders. Although literature reported a non-significant association between attachment avoidance and PGDS (Buur et al., 2024), some recent research highlighted some other pathways (Lenzo et al., 2022; Giunta et al., 2024). Thus, it is plausible to hypothesize that attachment avoidance showed a significant effect through mechanisms other than psychosomatic dysregulation—for example, such as poor reflective functioning (Giunta et al., 2024)—or increased physiological stress reactivity that remains unrecognized (Mikulincer and Shaver, 2019). Future research should investigate these complex pathways from attachment to grief reactions.

Findings from this study may have significant clinical implications, highlighting the need for tailored psychological interventions in order to increase therapeutic efficacy in PGD. For example, in individuals with attachment anxiety, characterized by an overactivation of the attachment system and difficulties in emotional regulation (Mikulincer and Shaver, 2019, 2022), approaches centered on the development of affective regulation strategies could be indicated, such as cognitive-behavioral therapy focused on grief and mindfulness techniques (Srivastava et al., 2025). Differently, in subjects with avoidant attachment, who tend to suppress relational needs and emotional states (Brennan et al., 1998; Mikulincer and Shaver, 2019), a gradual approach oriented toward mentalization and emotional awareness could be useful, such as psychodynamic therapy focused on grief or interventions based on mentalization-based therapy (Srivastava et al., 2025). These considerations highlight the significance of a differentiated and multimodal therapeutic approach that integrates both top-down and bottom-up components, while also considering attachment orientation as a clinically relevant variable.

Although this study offers insight into the relationship between attachment insecurity and PGDS, some limitations should be considered. Firstly, the cross-sectional design inherently restricts causal interpretations and precludes a comprehensive understanding of the potentially reciprocal dynamics underlying the observed associations. Psychosomatic dysregulation, for instance, may function both as a contributing factor to the intensification of PGD and as a process exacerbated by the emotional burden of prolonged grief. Secondly, some sample characteristics such as the predominance of female participants in both groups may introduce a sampling bias, which could affect the external validity and limit the generalizability of the results, especially for male bereaved. Thirdly, even though the time since loss was statistically controlled for in the mediation analyses, its potential residual influence cannot be entirely ruled out. Lastly, the use of self-report instruments to assess attachment, psychosomatic dysregulation, and PGDS (TGI-SR+, RQ, PDI), though validated and widely used, entails potential biases related to social desirability and self-perception.

In sum, this study points out that psychosomatic dysregulation partially mediates the association between attachment anxiety and PGDS, whereas no mediation was found for attachment avoidance, suggesting distinct underlying pathways. These findings highlight the central relevance of bodily process and emotional regulation in grief reactions. Psychological interventions that foster body–mind integration for bereaved individuals with attachment anxiety, independently by the type of loss, may be efficacy for PGDS.

## Data Availability

The original contributions presented in the study are included in the article/supplementary material, further inquiries can be directed to the corresponding author.
